# Anti-obesity drug discovery: advances and challenges

**DOI:** 10.1038/s41573-021-00337-8

**Published:** 2021-11-23

**Authors:** Timo D. Müller, Matthias Blüher, Matthias H. Tschöp, Richard D. DiMarchi

**Affiliations:** 1 Institute for Diabetes and Obesity, Helmholtz Diabetes Center, Helmholtz Zentrum München, Neuherberg, Germany; 2 German Center for Diabetes Research (DZD), Neuherberg, Germany; 3 Helmholtz Institute for Metabolic, Obesity and Vascular Research (HI-MAG) of the Helmholtz Zentrum München at the University of Leipzig and University Hospital Leipzig, Leipzig, Germany; 4 Helmholtz Zentrum München, Neuherberg, Germany; 5 Division of Metabolic Diseases, Department of Medicine, Technische Universität München, München, Germany; 6 Department of Chemistry, Indiana University, Bloomington, IN USA

**Keywords:** Obesity, Diabetes

## Abstract

Enormous progress has been made in the last half-century in the management of diseases closely integrated with excess body weight, such as hypertension, adult-onset diabetes and elevated cholesterol. However, the treatment of obesity itself has proven largely resistant to therapy, with anti-obesity medications (AOMs) often delivering insufficient efficacy and dubious safety. Here, we provide an overview of the history of AOM development, focusing on lessons learned and ongoing obstacles. Recent advances, including increased understanding of the molecular gut–brain communication, are inspiring the pursuit of next-generation AOMs that appear capable of safely achieving sizeable and sustained body weight loss.

## Abstract (web summary)

The development of therapies that are capable of safely achieving sizeable and sustained body weight loss has proved tremendously challenging. Here, Müller et al. provide an overview of the history of anti-obesity drug development, focusing on lessons learned, ongoing challenges and recent advances in the field.

## Introduction

Control of excess body fat is one of the greatest healthcare challenges of our time^1,2^. The global obesity prevalence has nearly tripled since 1975 and, within the United States, excess body weight afflicts more than two thirds of the population, with more than one third of adults and 20% of adolescents having obesity (see Related links).

**Fig. 1:**
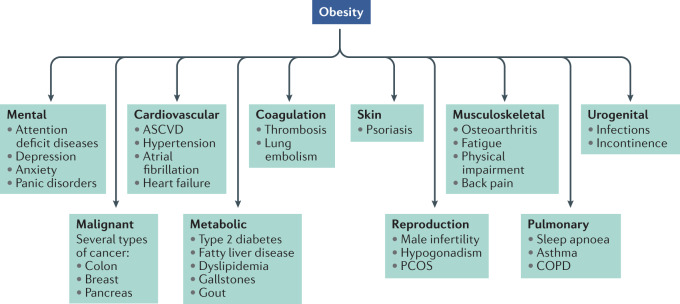
Obesity-associated metabolic disturbances. Most prominent metabolic and psychological comorbidities associated with morbid obesity. ASVCD, atherosclerotic cardiovascular disease; COPD, chronic obstructive pulmonary disease; PCOS, polycystic ovary syndrome.

Obesity promotes the incidence of conditions such as type 2 diabetes (T2D)^3^ and cardiovascular diseases (CVD)^4^, and increases the risk of death due to cancer of the oesophagus, colon and rectum, liver, gallbladder, pancreas and kidney^5–7^. It complicates the management of multiple diseases, enhancing the prospect for unfavourable outcomes, as prominently noted in the current COVID-19 pandemic^8^. Compared with normal weight, individuals with a body mass index (BMI) of 30–34.9 kg m^–2^ carry a hazard ratio for overall mortality that is elevated by more than 40% and at a BMI > 40 kg m^–2^ the relative rate increases to 100%^5^. It is estimated that 4–9% of all cancer diagnoses are attributable to excess body fat^9,10^, and that obesity correlates with poorer prognosis for multiple malignant diseases^6,7^. Obesity is associated with decreased life expectancy of 5–20 years depending upon its duration, the magnitude of excess weight and the emergence of associated comorbid diseases^5,11,12^. Starting early in life, obesity increases the prevalence for psychological, neurological, pulmonary, gastrointestinal, renal, musculoskeletal and endocrine diseases^13^ (Fig. 1). Estimates of the financial burden of obesity upon modern healthcare systems are sizeable, with more than US$190 billion spent annually in the United States alone for obesity-related illnesses^14^.

Commonly acknowledged environmental factors accounting for the steep increase in global obesity are increased access to energy-dense food coupled with reduced physical activity^15^. Sleep deprivation^16^, circadian desynchronization^17^, chronic stress^18^ and the use of anti-epileptic and psychotropic drugs^19^ may further propel weight gain. Genetic and environmental factors each appreciably contribute to the variance in BMI^20^. With an estimated heritability of ∼40–70%^20,21^, the contribution of genetic factors to BMI is comparable with that reported for Tourette syndrome (58–77%)^22^, psoriasis (66%)^23^, heart disease (34–53%)^24^ or breast cancer (25–56%)^25^.

Increased recognition of obesity as a chronic, degenerative disease^26,27^ serves to destigmatize the common belief that obesity results from insufficient self-discipline (see Related links). This further provides the framework for healthcare providers and insurance companies to establish obesity management programmes, promotes funding for basic and clinical research, and encourages pharmaceutical companies to develop strategies for body weight management. The central argument defining obesity as a chronic illness rather than a risk factor is the distinct pathophysiology that leads to excess fat accumulation and serves to defend it, coupled with homeostatic mechanisms that hinder weight loss and promote further weight gain^28^. These altered biological mechanisms may explain why short-term behavioural interventions are frequently insufficient for long-term weight loss.

As lifestyle and behavioural interventions provide moderate efficacy, obesity treatment strategies should be escalated by adding pharmacological and/or surgical interventions. Bariatric surgery represents the most effective approach to weight loss, leading to decreased mortality from CVD or cancer by 30% and 23%, respectively^29^. With steadily improving laparoscopic procedures, hospitalization time decreases and bariatric surgery increases overall life expectancy by as much as 3 years^29^, with notable and sustainable improvements in blood pressure, glucose and lipid metabolism^30^. Nonetheless, surgical interventions are incapable of meeting the global magnitude of medical need.

**Fig. 2:**
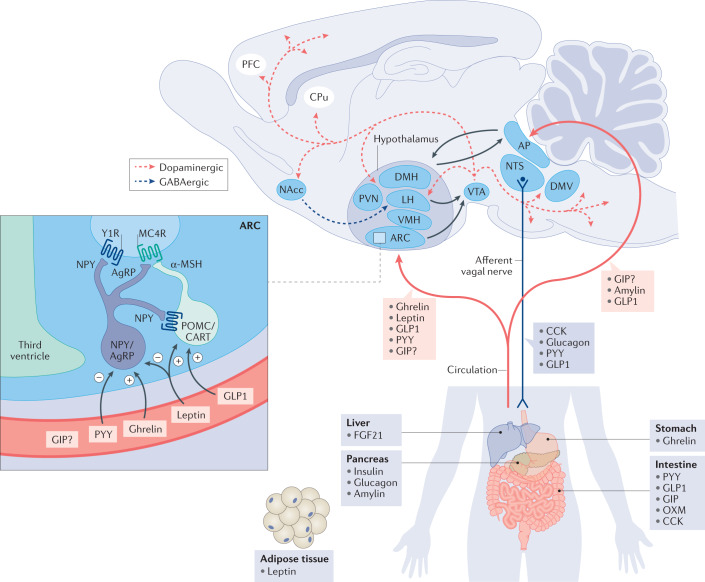
Gut–brain regulation of food intake. Peripheral hormones integrate in central control of homeostatic and hedonic eating behaviour. α-MSH, α-melanocyte-stimulating hormone; AgRP, agouti-related peptide; AP, area postrema; ARC, arcuate nucleus; CART, cocaine- and amphetamine-regulated transcript; CCK, cholescystokinin; CPu, caudate putamen; DMH, dorsomedial hypothalamus; DMV, dorsal motor nucleus of the vagus; FGF21, fibroblast growth factor 21; GIP, glucose-dependent insulinotropic polypeptide; GLP1, glucagon-like peptide 1; LH, lateral hypothalamus; MC4R, melanocortin 4 receptor; NAcc, nucleus accumbens; NPY, neuropeptide Y; NTS, nucleus tractus solitarius; OXM, oxyntomodulin; PFC, prefrontal cortex; POMC, pro-opiomelanocortin; PVN, paraventricular nucleus; PYY, peptide tyrosine tyrosine; VMH, ventromedial hypothalamus; VTA, ventral tegmental area; Y1R, neuropeptide Y receptor type 1.

The pursuit of anti-obesity medications (AOMs) has been tremendously challenging for technical and societal reasons. Only in the last two decades has the definition of the molecular mechanisms that control appetite (Box 1; Fig. 2) advanced to a point where drug discovery can be rationally pursued^31^. Historically, there has been a collection of AOM failures that have occurred after regulatory approval. Most of these pertain to adverse cardiovascular effects (sibutramine, fenfluramine, dexfenfluramine, rainbow pills), increased suicidal risk (rimonabant) or enhanced likelihood of drug dependence and abuse (methamphetamine) (Table 1). As such, certain drugs are recommended only for short-term use, due to addictive potential or emergence of tachyphylaxis (phentermine, amfepramone, cathin hydrochloride)^32,33^. Nonetheless, phentermine has not shown adverse cardiovascular outcomes in real-life studies and remains a commonly prescribed long-term AOM.

**Table 1:** History of weight loss drugs

Drug (full dose and administration)	Company	Approval	Weight loss (placebo/drug)	Side effects	Refs
***Mitochondrial uncoupler***					
DNP	Stanford University	1933–1938 (USA)	No data for controlled treatment ≥52 weeks	Hyperthermia, tachycardia, fever, tachypnoea, death	^34^
***Sympathicomimetic***					
Diethylpropion/afepramone	Merrell National Drug	1959–present (EU)	No controlled treatment ≥52 weeks	Nausea, constipation, insomnia, headache, tension and irritation, seizures	^34^
Methamphetamine	Abbott Laboratories	1947–1979 (USA)		Nigh risk for abusiveness and addiction	^34^
Phenmetrazine	Ciba-Geigy Corp	1956–present (USA)		Nausea, diarrhoea, dry mouth	^34^
Phendimetrazine	Carnick Laboratories	1959–present (USA)		Nausea, diarrhoea, dry mouth	^34^
Phenylpropanolamine	Thompson Medical	1960–2000 (USA)		Haemorrhagic stroke	
Fenfluramine and dexfenfluramine	Wyeth Ayerst	1973–1997 (USA)	−2.8%/−5.4%	Cardiac valvular insufficiency and pulmonary hypertension	^285^
Cathine (nor-pseudoephedrine) (53.3 mg, OD, oral)	Riemser Pharma	1975–present (EU, only for short-term use)	−2.4%/−6.6% to 9.9% (dose-dependent, short-term use only)	Tachycardia, increase in blood pressure, restlessness, sleep disorder, depression	^32^
Sibutramine (10 mg, OD)	Abbott Laboratories	1997–2010 (USA, EU)	+0.7%/−1.7%	Non-fatal myocardial infarction and stroke (in individuals with pre-existing CVD)	^154^
Phentermine (15–30 mg, OD, oral)	Teva Pharmaceuticals	1959–present (USA, only for short-term use)	−1.7%/−6.6% to −7.4% (dose-dependent)	Palpitations, elevated blood pressure	^286^
***Polypharmacy***					
Rainbow pills	Clark & Clark and others	1961–1968 (USA)	No controlled treatment ≥52 weeks	Insomnia, palpitations, anxiety, increase in heart rate and blood pressure, death	^287^
***CB1 receptor blocker***					
Rimonabant (20 mg, OD)	Sanofi SA	2006–2009 (EU)	−1.6%/−6.4%	Depression, suicidal ideation	^288^
***Pancreatic lipase inhibitor***					
Orlistat (120 mg TID, oral)	Roche Pharmaceuticals	1999–present (USA, EU)	−6.1%/−10.2%	Liver injury, gastrointestinal symptoms	^289^
***5-HT***_***2C***_ ***serotonin agonist***					
Lorcaserin (10 mg, BID, oral)	Arena Pharmaceuticals, Eisai	2012–2020 (USA)	−2.2%/−5.8%	Depression, suicidal ideation, palpitations, gastrointestinal symptoms, increased cancer risk	^65^
***Sympathomimetic/anticonvulsant***					
Phentermine/topiramate ER (with titration) (15 mg/92 mg, OD, oral)	Vivus	2012–present (USA)	−1.2%/−7.8% to 9.3% (dose-dependent)	Depression, suicidal ideation, cardiovascular events, memory loss, birth defects	^290,291^
***Opioid receptor antagonist/dopamine and noradrenaline reuptake inhibitor***					
Naltrexone SR/bupropion SR (with titration) (32 mg/360 mg, BID, oral)	Orexigen Therapeutics Inc.	2014–present (USA, EU)	−1.3%/−5.0% to −6.1% (dose-dependent)	Seizures, palpitations, transient blood pressure elevations	^292^
***GLP1R agonists***					
Liraglutide (with titration) (3.0 mg, OD, subcutaneous injection)	Novo Nordisk	2014–present (USA, EU)	−2.6%/−8%	Nausea/vomiting, diarrhoea, constipation, pancreatitis, gallstones	^176^
Semaglutide (2.4 mg, once weekly, subcutaneous injection)	Novo Nordisk	2021 (USA)	−2.4%/−14.9%	Nausea/vomiting, diarrhoea, constipation	^38^

BID, twice daily; CB1, cannabinoid receptor 1; CVD, cardiovascular disease; DNP, 2,4-dinitrophenol; ER, extended release; GLP1R, glucagon-like peptide 1 receptor; SR, sustained release; TID, three times daily; OD, once daily.

Until recently, long-term pharmacotherapy to achieve body weight normalization along with suitable tolerability and safety remained an insurmountable challenge^34^. However, recent clinical trials with advanced therapeutic candidates including glucagon-like peptide 1 receptor (GLP1R) agonism are promoting the belief that breakthrough, drug-based management of obesity may be possible. On 4 June 2021, the US Food and Drug Administration (FDA) approved semaglutide 2.4 mg for chronic weight management in adults with obesity or overweight with at least one weight-related condition (such as high blood pressure or cholesterol, or T2D), for use in addition to a reduced-calorie diet and increased physical activity (see Related links). This now constitutes the second GLP1R agonist registered for body weight management, as liraglutide 3 mg was approved by the FDA in 2014 for treatment of adult obesity and in 2020 for obesity in adolescents aged 12–17 years (see Related links).

**Fig. 3:**
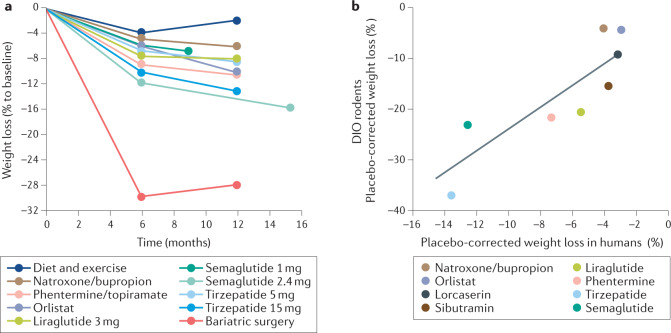
Body weight loss by AOMs in humans and rodents. Body weight loss achieved through lifestyle changes, currently approved anti-obesity medications (AOMs) and bariatric surgery (part **a**) and correlation of drug-induced body weight loss in rodents and humans (part **b**). Data in panel **a** refer to liraglutide 3 mg (ref.^176^), orlistat^289^, naltrexone/bupropion^292^, phentermine/topiramate^291^, semaglutide 1 mg (ref.^125^), semaglutide 2.4 mg (ref.^38^) and tirzepatide (5 and 15 mg)^126^. Data in panel **b** refer to naltrexone/bupropion^39,295^, orlistat^39,296^, lorcaserin^39,297^, sibutramine^154,298^, liraglutide^39,299^, phentermine^121,145^, semaglutide^38,123^ and tirzepatide^122,127^.

With the exception of semaglutide 2.4 mg (refs^35–38^), the average percent body weight reduction for currently registered drug treatments varies in the single-digit range, with only a small fraction of subjects capable of achieving and maintaining >10% loss at tolerable doses^39,40^ (Fig. 3). Although such weight loss is clinically meaningful^41,42^, and serves to improve the severity of comorbid diseases^43^, it is paltry when viewed against the efficacy of bariatric surgery^41,44^. An ideal AOM should sizeably and sustainably correct excess weight while reducing the risk of CVD and other comorbidities, devoid of the potential for abuse, tachyphylaxis and other adverse effects that have historically plagued this field^41^. It is a lofty goal and, at times, still challenged by the question of whether obesity itself constitutes a disease worthy of chronic drug therapy^45,46^.

This article reviews the history of obesity drug therapy and discusses ongoing challenges and recent advances in the development of AOMs. Although mechanistic understanding of energy homeostasis has dramatically progressed since the discovery of leptin just over 25 years ago^47^, the translation to targeted therapies has largely been empirical, with rodent models remaining of seminal importance, but of variable value for drug candidate selection. This is prominently witnessed in the ongoing debate pertaining to the gut hormone glucose-dependent insulinotropic polypeptide (GIP), where, based on rodent pharmacology studies, both GIPR agonism or antagonism can provide supplemental pharmacology to GLP1 agonism^48^. Lifelong pharmacological management of chronic diseases such as hypertension might offer relevant benchmarks for obesity treatment strategies. In these diseases, it is common practice to target multiple mechanisms to achieve optimal disease management. It seems inevitable, and with good precedent, that such a conceptual approach to lowering body weight will eventually prevail^40^.

Box 1 Endocrine control of food intake Hunger and satiety are controlled by a complex neuroendocrine system that depends on constant signal integration and bidirectional crosstalk between key feeding centres in the brain and the periphery (Fig. 2). Various food intake-regulating hormones are secreted by the gastrointestinal tract, the liver, the pancreas or the adipose tissue and they jointly act on the brain, in particular the hypothalamus and/or the hindbrain, to modulate appetite and satiety. Food intake-related gut hormones can be classified as short-term regulators of food intake, which are either secreted in anticipation of (ghrelin), response to (cholescystokinin (CCK), peptide tyrosine tyrosine (PYY), glucagon-like peptide 1 (GLP1), glucose-dependent insulinotropic polypeptide (GIP), oxyntomodulin (OXM)) or deprivation from (glucagon, fibroblast growth factor 21 (FGF21)) nutrients, and long-term regulators of food intake, which signal to the brain in proportion to the amount of fat stored in the body (leptin, insulin, amylin). Apart from homeostatic regulation of food intake, hunger and satiety are influenced by environmental factors such as palatability and food odour. Brain areas implicated in hedonic eating behaviour include those next to the hypothalamus and the brainstem, and also dopaminergic brain reward centres in the mesolimbic brain region as well as the hippocampus and cortex^300,301^.

The communication between the periphery and the brain is mediated through afferent fibres of the vagus nerve that project to, for example, the nucleus tractus solitarius (NTS) of the hindbrain, or via the circulation, which reaches the brain through the median eminence of the hypothalamus or the area postrema (AP) of the brainstem (Fig. 2). The hypothalamic melanocortinergic system represents a key hub in control of homeostatic food intake that comprises orexigenic neurons that co-express neuropeptide Y (NPY) and agouti-related peptide (AgRP) and anorexigenic neurons that co-express pro-opiomelanocortin (POMC) and cocaine- and amphetamine-regulated transcript (CART). Activation of NPY/AgRP neurons leads to secretion of AgRP, which stimulates food intake through blocking of the melanocortin 4 receptor (MC4R), whereas activation of Pomc/Cart neurons leads to secretion of α-melanocyte-stimulating hormone (α-MSH), which activates MC4R to inhibit food intake (Fig. 2).

The stomach-derived peptide hormone ghrelin reaches the hypothalamus via the median eminence and stimulates homeostatic food intake through activation of NPY/AgRP neurons^245^, while stimulating hedonic eating through activation of dopaminergic neurons in the ventral tegmental area^302^. To activate its receptor, ghrelin requires *N*-octanoylation (acylation) at its serine 3 residue, and as dietary lipids are used for ghrelin acylation, this suggests that ghrelin might also act as a nutrient sensor that informs the brain about incoming nutrients^245^.

Although best known for its ability to lower blood glucose, insulin was the first hormone demonstrated to rise in proportion to body fat and to decrease food intake via central nervous system (CNS) mechanisms^276,303,304^. Amylin is co-secreted with insulin from the pancreatic β-cells and decreases homeostatic food intake via signalling through the AP^242,305–308^. Amylin also affects hedonic eating behaviour via signalling through the mesolimbic dopamine system in the ventral tegmental area and the nucleus accumbens (NAcc)^233,309^.

FGF21 is secreted primarily from the liver under conditions of fasting, and decreases body weight by increasing energy expenditure via central and peripheral mechanisms^310–313^. CCK is secreted from intestinal I cells in response to nutrient (especially fat) ingestion. It binds to the CCK1 receptor (CCK1R) to decrease food intake through a reduction in meal size^314–316^. The CCK1R is widely expressed in vagal afferents, the NTS and the AP^317,318^, suggesting that CCK transmits the satiety signal via the vagus to the brainstem, from which the satiety signal is projected to the hypothalamus.

PYY is co-secreted with GLP1 from L cells of the distal bowel. Its major circulating form (PYY_3–36_) has been suggested to lower food intake through Y2 receptor-mediated inhibition of NPY/AgRP neurons, and hence activation of POMC neurons^278^. GLP1 decreases food intake via CNS mechanisms that seem to involve direct activation of POMC/CART neurons, but also activation of neurons in the AP and NTS^130^. GLP1R agonists also modulate hedonic food intake by acting on the dopaminergic brain reward system in the ventral tegmental area, NAcc and lateral septum^319–322^. Depending on the molecule and the route of administration, GLP1R agonists reach the hindbrain either via the circulation or through vagal afferents^130^. OXM exerts its anorexigenic action primarily through binding to the GLP1 receptor (GLP1R), and with lower affinity also binds to the glucagon receptor (GCGR)^323^. Glucagon decreases body weight through multiple mechanisms that include stimulation of lipolysis and energy expenditure and inhibition of food intake^323^. Glucagon suppression of food intake seems to be mediated via the liver–vagus–hypothalamus axis, as disconnecting the hepatic branch of the abdominal vagus is sufficient to block glucagon’s anorectic effect^323^. GIP regulation of energy metabolism remains enigmatic as activation and blocking of the GIPR receptor have both been shown to decrease body weight^48^. Recent studies suggest that GIP decreases food intake via CNS mechanisms^185,186^ and that GIP fails to affect food intake in mice with CNS loss of *Gipr*^185^.

## Regulation of body weight

Throughout human evolution, the environmental pressure for survival has likely included a drive to preserve body fat. With increased industrialization and ready access to high-fat foods, this acquired benefit has emerged as a liability. Physiologically, we defend body weight by peripheral and central mechanisms within a surprisingly small range, to protect against a broad array of conditions that include chronic overfeeding at one extreme and starvation at the other. Even the less well controlled long-term outcomes are associated with body weight change of rarely more than 20%, in either direction. The brain controls both hunger and systemic energy metabolism (Box 1; Fig. 2), and harbours most gene products and pathways that have been linked to obesity in hundreds of genetics studies^49–51^. However, direct modulation with central nervous system (CNS) signalling pathways requires selective targeting of cellular circuits, which remains a technological stretch, as historic attempts have shown more than once. For optimal weight loss efficacy, it seems apparent that drug therapy would have to target both energy intake and expenditure. However, intervention in central ‘survival’ mechanisms is a delicate endeavour that has led to withdrawal of many AOMs (Table 1). Striking a balance in striving for efficacy that promotes metabolic health and is psychologically meaningful to a patient, but of suitable chronic tolerability and safety, constitutes the medicinal challenge. Most currently registered medicines fulfil only a mere fraction of the performance that is desired, but there is reason for optimism as late-stage drug candidates hold much more promise^52,53^. A recurrent question is whether pharmacology can ever be as efficacious in lowering body fat at tolerable doses as bariatric surgery, or alternatively might it in time prove superior.

Unquestionably, advances in understanding the molecular elements that control appetite and energy utilization have provided a road map for more informed AOM development (Box 1; Fig. 2). The sizeable and rapid lowering of body weight achieved by bariatric surgery that results in much improved long-term mortality^29^ has further provided a vision of what might be pharmacologically possible. Indeed, mimicking the effects of bariatric surgery has become one vision for discovery of future AOMs.

## History of AOMs

Pharmacotherapy of obesity has a long and chequered history that is constituted by promising drugs that were withdrawn due to safety concerns (Box 2). In the last century, the pharmacological management of obesity has included amphetamines, thyroid hormones, dinitrophenol and various drug combinations (rainbow pills) that were withdrawn shortly after regulatory approval due to serious adverse effects^34^ (Table 1). Several centrally acting sympathomimetics such as phentermine, cathine and diethylpropion continue in short‐term use. Medicines that have been investigated in obesity include agents as diverse as mitochondrial uncouplers^54–56^, sympathomimetics^33,34^, serotonergic agonists^57–65^, lipase inhibitors^64,66^, cannabinoid receptor antagonists^67–69^ and a growing family of gastrointestinal-derived peptides chemically optimized for pharmaceutical use^34^. A sobering realization across most of these approaches is the common inability to achieve placebo-adjusted mean weight loss greater than 10% of initial body weight when chronically administered at tolerable doses. As greater weight loss is achieved, it is typically accompanied by various serious acute or chronic adverse effects^34^ (Table 1). A notable exception is the recently approved GLP1R agonist semaglutide 2.4 mg, which in phase III clinical trials decreased body weight in individuals with obesity or overweight without diabetes after 68 weeks of treatment by −14.9% relative to −2.4% in placebo-treated controls^38^.

AOMs predominantly function by peripheral or central pathways governing energy balance, but rarely both^70,71^. Orlistat, for example, acts as a lipase inhibitor to reduce the uptake of dietary fat from the gastrointestinal tract. AOMs that act centrally to increase satiety often function by modulating serotonergic, noradrenergic or dopaminergic action. These AOMs block catecholamine reuptake or directly stimulate satiety receptors in the hypothalamus and limbic system^34^. In addition, certain AOMs increase energy expenditure by inducing thermogenesis or lipolysis through actions at peripheral or central sites^34^. Sympathomimetic agents, such as phentermine, operate in the CNS, where they increase norepinephrine in the synaptic cleft and directly stimulate β-adrenergic receptors. The sympathomimetic agent phentermine has been combined with topiramate, an anti-epileptic carbonic anhydrase inhibitor that potentially affects energy metabolism through modulation of GABAergic neurotransmission^72^. Sympathomimetics may also increase thermogenesis^73^, but α‐adrenergic and β‐adrenergic receptor stimulation is also associated with vasoconstriction and increased sympathetic tone that can result in increased blood pressure and heart rate.

Despite prominent failures of AOMs (Box 2), more recently approved drugs for obesity management are accessible for use in addition to behaviour modifications. In the United States and Europe, orlistat, naltrexone/bupropion, liraglutide 3 mg and, most recently, semaglutide 2.4 mg are registered and promoted. In addition, in the United States, phentermine/topiramate is even available for long-term use^40^.

Bupropion is a reuptake inhibitor of dopamine and norepinephrine. Although naltrexone, an opioid antagonist, does not cause weight loss in monotherapy, it blocks the inhibitory effects of opioid receptors activated by β-endorphin released in the hypothalamus, which stimulates feeding. In combination with bupropion, it reduces food intake^74^. Although naltrexone/bupropion may increase blood pressure and should therefore not be used in patients with uncontrolled hypertension, no adverse signal for increased cardiovascular events was found in the interim analysis of a cardiovascular outcome trial^75^.

In 2014, liraglutide 3 mg became the first GLP1-based AOM to be introduced to the US market for treatment of obesity in adults, and in 2020 was approved for weight management in adolescents aged 12 years and older with obesity (see Related links). Prior to this (since 2010), liraglutide was used as a subcutaneous injection for treatment of T2D in daily doses of up to 1.8 mg, demonstrating a lower incidence of major adverse cardiovascular events compared with best standard of care in the LEADER trial^76^. The most common complaints in patients treated with subcutaneous liraglutide 1.8 mg are gastrointestinal side effects including nausea, diarrhoea, vomiting and constipation^77^. The more recently FDA-approved semaglutide at a dose of 2.4 mg lowers mean body weight to ~15% after 68 weeks of treatment (relative to ~2.4% in placebo controls)^38^. The drug is generally well tolerated although the typical GLP1-related adverse effects (primarily nausea, diarrhoea, vomiting and constipation) still prevail^38^.

Box 2 Prominent failures of AOMs A prominent example of a promising anti-obesity medication (AOM) that ended poorly is the appetite suppressant fenfluramine. It received US Food and Drug Administration (FDA) approval in 1996, but was terminated a year later due to adverse effects. The specific d-stereoisomer (dexfenfluramine) was US registered in 1996 under the name Redux but was terminated just a year later. Both drugs stimulate the release of 5-hydroxytryptamine (5-HT; also known as serotonin) and inhibit its reuptake in the synaptic cleft. Dexfenfluramine was purported to be more selective in biological action, with fewer adverse effects than fenfluramine. Several randomized, controlled trials demonstrated significant weight loss with either agent, or in particular when combined with phentermine^59,324–326^. However, these benefits were accompanied by concerns for valvular heart disease and primary pulmonary hypertension (PPH). A meta-analysis of observational studies reported that one in eight patients treated for more than 90 days with fenfluramine demonstrated valvular regurgitation^327^. These adverse events were mechanistically linked to direct stimulation of 5-HT_2B_ receptors on the interstitial cells of the mitral and aortic valves and were similar to observations in patients with carcinoid tumours or excessive exposure to ergot. The use of dexfenfluramine and fenfluramine was also associated with an increased risk for PPH^150–152,328,329^.

Lorcaserin is a 5-HT_2C_ receptor agonist with much reduced affinity for other serotonergic receptors. The enhanced selectivity for the 5-HT_2C_ receptor was designed to improve the safety profile relative to less selective fenfluramine to lower the risk for PPH. Although lorcaserin is well tolerated, there are no long-term cardiovascular safety studies^65^. The drug should not be given with monoamine oxidase inhibitors, serotonin reuptake inhibitors, serotonin–norepinephrine reuptake inhibitors or other serotonergic drugs^40^. In 2020, the FDA requested withdrawal of lorcaserin due to clinical trials showing an increased occurrence of cancer (see Related links). However, at the same time the FDA approved lorcaserin for the treatment of chronic severe epilepsy in children (Dravet syndrome). Despite the inherent challenges to this specific approach, the pursuit for improved serotonergics is embodied by tesofensine, which is a multimode inhibitor of norepinephrine, serotonin and dopamine reuptake that was initially advanced for treatment of Alzheimer disease. In a phase II study, it was reported to dose-dependently decrease body weight by 4.4–10.4%^166,330^. Tesofensine also improved LDL cholesterol and triglyceride levels, but led to increased heart rate. It is difficult to determine the current development of the drug candidate as there are few peer-reviewed reports and the commercial sponsor has changed more than once^166^.

Another prominent failure of an AOM was sibutramine — a norepinephrine and serotonin reuptake inhibitor that reduces appetite and promotes thermogenesis. Sibutramine was approved by the FDA in 1997 but was withdrawn due to increasing the risk of cardiovascular events in a high-risk population for which sibutramine’s use was originally not intended^154^. The increase in sympathetic activity enhances blood pressure and heart rate, and as such, sibutramine was contraindicated in patients with a history of coronary artery disease, heart failure, tachycardia, peripheral arterial occlusive disease, arrhythmia, cerebrovascular disease or inadequately controlled hypertension. To address the potential for adverse cardiovascular events, the SCOUT trial was initiated to determine long-term cardiovascular outcomes in a high-risk population. More than 10,000 patients with overweight or obesity, combined with pre-existing cardiovascular disease (CVD) and/or type 2 diabetes (T2D), were treated with the aim of reducing the primary composite outcome of non-fatal myocardial infarction, non-fatal stroke and resuscitation after cardiac arrest or cardiovascular death. Alarmingly, the incidence of non-fatal myocardial infarction and non-fatal stroke was significantly higher in patients treated with sibutramine^156,331^, although other studies suggested that sibutramine is fairly safe in patients without higher risk for a cardiovascular event^153,154,332^. Although cardiovascular safety concerns terminated further use of sibutramine, fenfluramine and phenylpropanolamine, a struggle with adverse psychological effects emerged elsewhere. One prominent example here is rimonabant, an endocannabinoid 1 receptor (CB1) antagonist shown to decrease appetite, enhance thermogenesis and diminish lipogenesis preclinically and in numerous human trials^333^. Upon emerging reports of suicidal ideation and serious depression, the FDA rejected its registration in 2007 (ref.^334^).

## Challenges confronting AOM development

### Heterogeneity of patient cohorts

Obesity is a heterogeneous condition constituted by rare monogenetic^49,78^ and, more commonly, polygenic aetiology associated with neurobehavioural, endocrine and metabolic causes^51,79–86^. Obesity-related risk factors and/or quantitative trait loci are found on nearly every chromosome^87–90^. Epigenetic processes may yet account for additional factors predisposing to obesity^91–93^. Further scientific dissection of the heterogeneity in genetic, epigenetic and environmental risk factors is of major importance as these may not only explain the variance in BMI but also affect the individual response to certain pharmacotherapies^82,94^.

Rare chromosomal abnormalities are observed in >10% of children with severe obesity^51^. Monogenetic obesity is observed in individuals with loss-of-function mutations in genes encoding for leptin^95–97^, the leptin receptor (LEPR)^98^, pro-opiomelanocortin (POMC)^99^ and the melanocortin 4 receptor (MC4R)^50,100^. The most common polygenic risk factors for obesity include mutations in the fat mass and obesity-associated gene (FTO)^101^ and MC4R^102^.

A more thorough metabolic and genetic characterization in combination with detailed disease aetiology and response to different mechanisms in drug action should lead to an improvement in patient care. Additionally, this can also potentially foster the next generation of AOMs by advancing a deeper understanding into the molecular pharmacology of body weight regulation. It remains to be determined whether one, two or more mechanisms in drug action will prove successful in treatment of most patients with obesity, or whether far more diverse customization will be required to optimally tackle the obesity pandemic.

### Neuroendocrine considerations

Various peripherally derived endocrine factors regulate food intake by jointly acting on defined neurocircuits in the hypothalamus and other brain regions^103–106^ (Box 1; Fig. 2). Although this tightly controlled system is pivotal for survival, it has emerged as a major obstacle to achieving sizeable body weight reduction, as it progressively defends against negative energy balance and undernutrition^107–110^. One of the likely relevant underlying mechanisms is a decrease in peripheral adiposity signals (leptin, insulin) following weight loss, and prolonged fasting leads to increased expression and sensitization to orexigenic neuropeptides in the hypothalamus and the hindbrain. Simultaneously, the expression of and sensitivity to anorexigenic neuropeptides decrease in these same areas to constitute a double-barrelled defence of body weight^111–113^. Concurrently, the density and strength of the orexigenic agouti-related peptide (AgRP)/neuropeptide Y (NPY) fibres that project from the arcuate nucleus (ARC) to the paraventricular hypothalamic nuclei increase in response to prolonged fasting. This remodelling of the ARC^AgRP/NPY^ projections correlates with increased activation of paraventricular hypothalamic nuclei neurons with the goal to restore food intake^114^. Another obstacle in weight loss pharmacology is that persistent elevation of adiposity signals such as leptin and insulin results in desensitization, leading to an impaired responsiveness of this homeostatic system^115–117^. A striking finding supporting this perspective is that leptin supplementation shows remarkable efficacy in lowering body weight in individuals with congenital leptin deficiency^96,118,119^, but is largely ineffective in more common polygenetic forms of obesity^115–117^.

### Translation of pharmacology from animals to humans

#### Effects on body weight

Several studies have shown high correlation between rodents and humans in the weight-lowering properties of phentermine/topiramate, sibutramine, rimonabant, topiramate, phentermine and orlistat^120,121^. Meta-analyses confirmed that results from animal models predicted human effects of the more recently approved naltrexone/bupropion^39^. Incretin-based therapy with peptides such as exendin 4, liraglutide, semaglutide and the GIP/GLP1 dual agonist tirzepatide lower body weight in rodents^122,123^ and humans^38,53,124^. Overall, with the exception of semaglutide 2.4 mg (ref.^38^), the mean placebo-corrected body weight loss achievable through therapy with a registered AOM resides in a relatively narrow range of 3–7% after 6–12 months of treatment, with a finite fraction of subjects surpassing weight loss of 10% and much fewer 15% relative to placebo^39,41^ (Fig. 3). Of special merit, semaglutide 2.4 mg and tirzepatide (10 or 15 mg once weekly) have recently reported a mean weight loss >10% in phase II and III clinical studies of subjects without diabetes^38,53,124–127^. Weight loss is considerably lower in patients with T2D, indicating that insulin resistance and chronic hyperglycaemia correlate with diminished efficacy of GLP1R agonists^35–38^.

However, whereas weight loss effects generally translate from rodents to humans, maximal efficacy is historically two to four times lower in humans relative to rodents (Fig. 3). It can be argued that greater relative weight loss in rodents is expected as mice possess a higher mass-specific energy expenditure than humans, with a greater contribution of brown adipose tissue to metabolic rate^128^. Consequently, mice may be more susceptible to drugs that affect energy expenditure. The high mass-specific metabolic rate requires sufficiently high caloric intake to protect against a chronic deficit in energy balance. It is consequently logical that mice can ingest food matching more than 10% of their body weight in a single day. Therefore, pharmacological inhibition of food intake offers a larger dynamic range and more immediate impact on weight loss in rodents relative to humans.

#### Glucose and lipid metabolism

A decrease in body weight of 5–10% can provide a clinically meaningful improvement in HbA_1c_, blood pressure, serum triglycerides and HDL cholesterol. These cardiometabolic improvements are progressively enhanced with further weight loss^129^. Decreased abdominal and hepatic fat deposition with improvement of β-cell function and insulin sensitivity are observed with modest degrees of weight loss. Certain AOMs are also capable of directly improving glycaemic control, which provides supplemental benefit to cardiometabolic outcomes. In particular, GLP1R and GIPR agonists improve glycaemia via their ability to enhance insulin secretion^130^ and by inhibiting gastric emptying to slow glucose entry to general circulation^131^.

In a large-scale meta-analysis comprising 29,018 participants, low to moderate improvement of glucose metabolism was demonstrated after 1 year of treatment with orlistat, naltrexone/bupropion, phentermine/topiramate and liraglutide^132^. All of these medicines also provided low to moderately improved LDL cholesterol and, except for orlistat, increased HDL cholesterol^132^. A recent placebo-controlled 26-week phase II study of tirzepatide dramatically improved HbA_1c_, fasting blood glucose and triglycerides with superior efficacy relative to treatment with the GLP1R selective agonist dulaglutide^124^. In phase III clinical trials, tirzepatide, at all tested doses, lowered HbA_1c_, fasting glucose and body weight with greater efficacy relative to a 1 mg dose of semaglutide^125^. Remarkably, 40 weeks of treatment with tirzepatide reduced HbA_1c_ <5.7% in 29–51% of patients depending on the dose, relative to 20% in patients treated with semaglutide 1 mg. Weight loss ≥15% was observed in 15–40% of patients treated with tirzepatide relative to 9% of patients treated with semaglutide^125^.

Curiously, not all weight-lowering agents improve glycaemia. In particular, fibroblast growth factor 21 (FGF21) agonists have proven enigmatic. As a class, they potently lower body weight and improve metabolism in preclinical studies. As one example of several FGF21 analogues clinically tested, the specific FGF21 agonist PF-05231023 demonstrated improvements in body weight, lipid metabolism and glycaemia in rodent and non-human primates^133,134^. Although there was a large and highly significant improvement in lipid metabolism in humans, PF-05231023 failed to improve glucose to any appreciable degree^134,135^. The effect on body weight is less certain, but considerably less than what has been preclinically reported or, certainly, in comparison with incretin-based therapy. Longer duration studies, or further increases in dose, may lead to clinically significant weight reduction or improvements in glucose metabolism of the type witnessed prominently in mice, but this remains to be demonstrated. However, at this point, it serves as a notable example where the pharmacological profile observed in preclinical studies has proven disappointingly different in clinical study.

### Safety aspects

The search for greater efficacy in next-generation AOMs must inevitably be anchored by the critical challenge of safety. Whether employing well-understood and more specific mechanisms of action, or pursued through adjunctive agents proven to be independently safe, the risk for toxicity must be fully assessed. To overcome this challenge, AOM development strategies need to increasingly reflect the heterogeneity of the human condition where diversity is far greater than can be encompassed in animal models. Initial AOM development and registration studies are influenced by commercial considerations, and as such specific patient populations, often of greatest need and risk, are under-represented. Clinical studies assessing different drug candidates are typically more alike than different and are directed at large patient populations of common severity, typically individuals who are middle-aged with a body weight near to or slightly above 100 kg.

Undoubtedly, patients with extreme obesity, patients with multiple comorbidities and those at younger age confronting a lifelong struggle with excess body weight require special attention. In these instances, the importance of safety is paramount and yet the need for efficacy is equally enhanced. Certain AOMs unsuitable for the broader population with obesity might still hold promise in special circumstances and when carefully administered and monitored by a specialist. As an example, therapy with leptin in patients with congenital deficiency or with setmelanotide in patients deficient in POMC is highly effective^82,117,136^, yet currently of little (leptin) or uncertain (setmelanotide) value in other more common forms of obesity^115,116,137,138^. In any case of weight loss pharmacotherapy, the initial priority should be to safely achieve maximal weight reduction, followed by sustained therapy with AOMs and lifestyle changes that might require less supervision to maintain reduced body weight. Such an approach aims to reduce the risks of intensified therapy by scheduled migration to less forceful forms of therapy. Aggressive use of glucocorticoid therapy in severe inflammatory diseases followed by dose reduction seems a suitable example, where careful patient management and specific drugs can suitably provide efficacy and safety^139^. Each patient managed by an informed caregiver might progress through a schedule of different drugs in combination with lifestyle modification to eventually achieve an optimal outcome.

Most obesity-related deaths are due to CVD^1,140^, and therefore improving cardiovascular health constitutes a primary objective for weight loss therapies. Although the risk of a major adverse cardiovascular event is generally lower in individuals who are lean relative to individuals with obesity^4^, the manner in which body weight is reduced by pharmacotherapy can result in significantly different outcomes, with some lessening and some worsening cardiovascular health. The cosmetic appeal for reduced body weight constitutes an independent risk for abuse as subjects strive for more rapid and larger reductions despite the potential for harmful effects. Importantly, there are no prospective cardiovascular outcome trial results for patients with obesity devoid of significant cardiometabolic comorbidities. The SELECT trial, designed to assess major adverse cardiovascular event reduction for selected AOMs, will clarify whether targeting obesity may result in improved cardiovascular outcomes^141^.

Amphetamine-induced release of norepinephrine can result in increased blood pressure, heart rate, cardiac contractility, conduction velocity and cardiac excitability via binding to vasculature and heart adrenergic receptors^142^. Amphetamines also carry a certain risk for abusiveness due to their action on the brain reward system. Cardiovascular effects ensuing from amphetamine abuse can present as chest pain, tachycardia, dyspnoea, primary pulmonary hypertension (PPH), dysrhythmias, acute myocardial infarction and, even, sudden cardiac arrest^142^. The fears of such toxicity led to discontinuation of methamphetamine (desoxyephedrine) for lowering body weight in the 1940s^34^. Phentermine and diethylpropion were designed to retain the anorectic activity, but with much reduced effects on the cardiovascular and brain reward system^143^. Several clinical studies report the absence of major adverse effects of phentermine^144,145^ or diethylpropion^146–149^ on blood pressure and heart rate. Nonetheless, rare occurrences of PPH and/or valvular heart disease have been reported and, therefore, their use is contraindicated in patients who are hypertensive or otherwise elevated in risk for CVD (see Related links). Distribution of fenfluramine and dexfenfluramine was discontinued in 1997 due to the risk of PPH and valvular heart disease^150–152^, whereas sibutramine was withdrawn due to the increased risk of stroke and non-fatal myocardial infarction, particularly in patients with CVD^142,153–155^ (see Box 2). Sibutramine has been associated with increased pulse rate^156^, blood pressure^157–160^ and cardiac arrhythmia^154,159^. Improvement in blood pressure has been reported in a meta-analysis for naltrexone/bupropion and orlistat, with greater cardiovascular beneficial effects reported for phentermine/topiramate^132^. Better yet, liraglutide (1.8 mg once daily)^76^ and injectable semaglutide 1 mg (ref.^161^) have been documented to improve cardiovascular outcomes in patients with T2D, notably with decreased rates of cardiovascular death, non-fatal myocardial infarction and non-fatal stroke.

An important question is why so many agents designed to decrease food intake eventually failed in clinical trials due to insufficient cardiovascular safety. The most common responses include species-related differences and the lack of preclinical models that reliably predict human cardiovascular safety. Although rodents and other animals are an essential tool to study drug effects on body weight and glucose control^162^, they are relatively resistant to adverse drug effects pertaining to the cardiovascular and pulmonary systems, rendering them less capable of predicting human cardiovascular safety. To date, there are no animal models that can predict drug-induced PPH and valvulopathy in humans^163^. Additionally, it is difficult to capture in otherwise healthy, inbred animals the heterogeneity of subjects that constitute human use. Most patients with obesity are older, afflicted with cardiovascular and associated diseases such as diabetes. It is near impossible to preclinically capture the full risk for use of AOMs in such a diverse patient population. Cardiovascular outcome trials such as the SELECT trial are needed to evaluate cardiovascular safety and potential cardiovascular risk reduction in patients with obesity but without major cardiovascular risk factors. The prominent factors that have collectively contributed to drug failure due to adverse cardiovascular effects have made themselves known in such clinical studies. The increased awareness has led to an emphasis on cardiovascular pharmacology and a demonstration for favourable cardiovascular outcomes as part of the process in AOM approval and broader distribution.

## Novel and emerging obesity therapies

Despite numerous disappointments, several prominent therapeutic targets have captured the attention of the scientific community^34,164–166^ (Table 2). They reflect the state of the art in how novel drug candidates have been identified and advanced to human study. Four target areas (leptin, ghrelin, mitochondrial uncouplers and growth differentiation factor 15 (GDF15)) were initiated and advanced with obesity constituting the primary therapeutic purpose (Table 2). By contrast, the research pertaining to incretins and, most notably, GLP1, as well as amylin, was predominately focused on diabetes that evolved through concurrent empirical observations of body weight lowering. However, the maturation of incretin biology has led to late-phase AOM candidates that potently activate GLP1R and/or GIPR to establish a much elevated, new benchmark for performance. These subjects are discussed in the following subsections.

**Table 2:** Weight loss drugs in clinical development

Agent	Company	Development stage	Indication	ClinicalTrials.gov ID/ref.^a^
***GLP1/glucagon dual agonists***				
Cotadutide (MEDI0382)	AstraZeneca	Phase II	T2D, NASH	NCT04019561 NCT03235050
BI 456906	Boehringer Ingelheim	Phase II	Obesity, T2D	NCT04153929
Efinopegdutide (^LAPS^GLP/GCG)	Hanmi Pharmaceutical	Phase II	NASH	NCT03486392
OXM	Eli Lilly	Phase I	T2D	See Related links
***GIP/GLP1 dual agonists***				
Tirzepatide	Eli Lilly	Phase III	Obesity, T2D	NCT04657003
GIP/GLP peptide I	Eli Lilly	Phase I	T2D	See Related links
GIP/GLP peptide II	Eli Lilly	Phase I	T2D	See Related links
NN9709	Novo Nordisk	Discontinued	Obesity, T2D	See Related links
***GIP/GLP1/glucagon tri-agonists***				
HM15211 (^LAPS^Triple Agonist)	Hanmi Pharmaceutical	Phase II	NASH	NCT04505436
GGG tri-agonist	Eli Lilly	Phase I	T2D	See Related links
NN9423	Novo Nordisk	Discontinued	Obesity, T2D	See Related links
***GIPR agonists***				
GIPR agonist long acting	Eli Lilly	Phase I	T2D	See Related links
ZP 6590	Zealand Pharma	Preclinical	Obesity	See Related links
***GLP1R agonists***				
Efpeglenatide (^LAPS^Exd4 Analog)	Hanmi Pharmaceutical	Phase III	T2D	NCT03353350 NCT03496298
Rybelsus	Novo Nordisk	Phase III	Obesity	NCT03919929
Danuglipron (PF-06882961)	Pfizer	Phase II	Obesity, T2D	NCT04707313 NCT03985293
GLPR-NPA	Eli Lilly	Phase I	T2D	See Related links
PF-07081532	Pfizer	Phase I	T2D	NCT04305587
***Glucagon analogue***				
HM15136 (^LAPS^Glucagon Analog)	Hanmi Pharmaceutical	Phase I	Obesity	See Related links
***Leptin sensitizers***				
Withaferin A	Academic, non-commercial	Phase I	Obesity, T2D	^293^
Celastrol	Academic, non-commercial	Preclinical	Obesity, T2D	^294^
Leptin/amylin	Amylin Pharmaceuticals	Discontinued	Obesity, T2D	See Related links
***Y2R agonists***				
PYY analogue	Eli Lilly	Phase I	T2D	See Related links
NN9748 (NN9747)	Novo Nordisk	Phase I	Obesity, T2D	NCT03574584
NNC0165-1875 + semaglutide	Novo Nordisk	Phase II	Obesity, T2D	NCT04969939
***Amylin/calcitonin dual agonists***				
KBP-089	Nordic Biosciences	Phase I	T2D	NCT03907202
KBP-042	Nordic Biosciences	Discontinued	T2D	NCT03230786
Davalintide	Amylin Pharmaceuticals	Discontinued	Obesity, T2D	See Related links
***Amylin analogues***				
Cagrilintide	Novo Nordisk	Phase II	Obesity, T2D	NCT04940078 NCT04982575
ZP 8396	Zealand Pharma	Preclinical	Obesity	See Related links
***Drugs targeting the ghrelin pathway***				
CYT009-GhrQb	Cytos Biotechnology	Phase I	Obesity	See Related links
Nox-B11	Noxxon Pharma	Preclinical	Obesity	See Related links
AZP-531	Millendo Therapeutics SAS	Discontinued	Hyperphagia in patients with Prader–Willi syndrome	NCT03790865
***Mitochondrial uncoupler***				
BAM15	Continuum Biosciences	Preclinical	Obesity, NASH	See Related links
***Other appetite suppressants***				
GDF15 (LA-GFD15)	Novo Nordisk	Phase I	Obesity	See Related links
LY-3463251 (GDF15 agonist)	Lilly	Phase I	T2D, obesity	NCT03764774
JNJ-9090/CIN-109 (GDF15 agonist)	Jansenn/CinFina Pharma	Phase I	Obesity	NA

GDF15, growth differentiation factor 15; GIP, glucose-dependent insulinotropic polypeptide; GLP1, glucagon-like peptide 1; GLP1R, GLP1 receptor; NA, not applicable; NASH, nonalcoholic steatohepatitis; OXM, oxyntomodulin; PYY, peptide tyrosine tyrosine; T2D, type 2 diabetes; Y2R, neuropeptide Y receptor type 2. ^a^See Related links for further information.

### Incretin-based therapy

#### GLP1-related drug candidates

Advancement in incretin biology over the last decades has resulted in a family of registered GLP1R agonists^167^. Their development was partially triggered by the success of oral DPP4 inhibitors that indirectly raise circulating concentrations of endogenous GLP1 and GIP to improve glycaemic control without risk of hypoglycaemia^168–174^. The parenteral administration of bioactive hormone paralogs and synthetic analogues provided increased circulating drug concentrations that resulted in enhanced glycaemic control and an increased appreciation for the inherent body weight-lowering properties of GLP1R agonism.

**Fig. 4:**
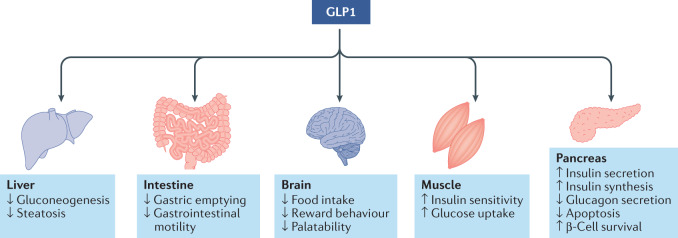
Regulation of body weight and glucose metabolism by GLP1R agonism. Glucagon-like peptide 1 receptor (GLP1R) agonism exerts both direct and indirect effects on energy and glucose metabolism in key peripheral organs as well as the brain.

The magnitude of weight lowering in initial clinical studies employing GLP1R agonists was modest and largely consistent with that previously observed with other gut hormones^175^. The pharmacology reports were associated with profound effects on gastrointestinal motility that complicated the assessment of how much of the weight lowering was a function of adverse local gastrointestinal effects that served to minimize appetite. The emergence of peptide analogues that extended and flattened pharmacokinetics in concert with dose titrations that lessened the frequency of adverse gastrointestinal effects, collectively enabled more sustained and intense treatment that solidified the metabolic and weight-lowering effects of GLP1R agonism. The specific mechanism of action is multifactorial, with gut, brain and systemic improvements in insulin sensitivity each contributing a finite fraction to the total efficacy^123^ (Fig. 4).

At the end of 2014, liraglutide 3 mg became the first GLP1R agonist to be approved for the treatment of obesity, at approximately twice the highest dose employed in the treatment of T2D. Following 1 year of treatment there was a reported mean decrease of 8% body weight in subjects treated with liraglutide relative to 2.6% in vehicle-treated controls^176^, with approximately two thirds of patients treated with liraglutide achieving >5% body weight reduction and one third experiencing >10%. This compares favourably with 27% and 11%, respectively, achieving similar outcomes in control subjects. The reduced body weight was associated with improvements in insulin sensitivity, circulating lipids and blood pressure, but with mean heart rate increasing by 2.4 bpm. The ability of liraglutide to lower body weight is competitive in magnitude to other conventional oral AOMs^165^. These results established that GLP1R agonism could be used to improve metabolism and modestly lower body weight in patients with obesity while reducing cardiovascular risk, building upon the previously demonstrated success in improving cardiovascular outcomes in chronic treatment of T2D (refs^76,177^). Whether liraglutide also improves cardiovascular outcomes in obesity without T2D warrants clarification. In addition, questions remain as to whether the degree of weight loss justifies the financial cost of the drug and regarding the obstacles associated with achieving compliance to a chronic, daily injection.

Semaglutide at 2.4 mg, a dose much higher than registered for treatment of T2D, gained approval in June 2021 for chronic weight management in adults with obesity or overweight. In a 1-year phase II study employing daily doses ~10% that of high-dose liraglutide, body weight loss was approximately doubled^53^. Daily dosing achieved >15% weight loss in half of the study participants, whereas one third experienced more than a 20% reduction. In a recent phase III clinical trial in patients of excess weight without diabetes, once-weekly treatment with semaglutide 2.4 mg decreased body weight after 68 weeks of treatment by −14.9% relative to −2.4% in placebo-treated controls^38^. In patients with diabetes and obesity, semaglutide decreased body weight by −9.6% relative to −3.4% in placebo controls^35^. These transformative results, particularly in the patients with obesity without diabetes, establish a new benchmark for efficacy and the FDA recently approved semaglutide 2.4 mg for the treatment of obesity as an adjunct to caloric restriction and increased physical activity (see Related links). Not long ago, achievement of this degree of weight loss was thought not to be safely possible, and weekly administration of semaglutide 2.4 mg is a notable virtue relative to liraglutide 3 mg, just as it proved in the treatment of T2D relative to liraglutide 1.8 mg. Whether comparable weight reduction can eventually be achieved by administration of the recently introduced oral form of semaglutide remains to be demonstrated^178^.

Several other peptide and small-molecule GLP1R agonists are currently in clinical development, including formulations designed for oral administration. Another oral GLP1R agonist (GLPR-NPA) is currently in phase II clinical trials at Eli Lilly (Table 2) (see Related links).

#### GIP-related drug candidates

Engagement of GIPR agonism for the treatment of obesity and T2D is regarded with notable scepticism, as the insulinotropic effect of GIP is diminished in patients with T2D^179^. In addition, appreciable preclinical evidence indicates that GIPR antagonism can improve systemic energy and glucose metabolism^180–183^, possibly through improvement of central leptin sensitivity^180^. However, long-acting (acyl) GIPR agonists decrease body weight in obese wild-type and GLP1R knockout mice^184,185^ and GIP affects body weight through signalling via the GIPR in the CNS. In line with this notion, GIPR is expressed in neurons of the hypothalamus and the hindbrain^186,187^ and DREADD-mediated activation of hypothalamic GIPR cells decreases food intake^186^. Consistent with this, single central administration of a fatty acyl-GIP decreases body weight and food intake in DIO mice and increases cFOS neuronal activity in the hypothalamus^185^. When peripherally administered, fatty acyl-GIP decreases body weight and food intake in obese wild-type and GLP1R knockout mice, but shows blunted weight loss in CNS GIPR-deficient mice^185^. In summary, long-acting GIPR agonists have been shown to decrease body weight and to improve glucose handling in a series of preclinical studies^184,185^ and a long-acting GIPR agonist is in phase I clinical trials for the treatment of T2D (Table 2) (see Related links).

#### Incretin-based poly-agonists

Simultaneous to the structural optimization of selective GLP1R and GIPR mono-agonists has been research to pharmacologically harness the fact that mammalian organisms govern energy balance through much more than a single hormone. The most notable breakthrough in that direction has been the discovery of poly-agonists that simultaneously target the GLP1, GIP and/or glucagon receptors^188,189^. Multiple drug candidates have advanced to clinical development (Table 2). The most prominent approaches pertain to unimolecular combination of GIP and/or glucagon receptor (GcgR) agonism with highly potent, complementary GLP1R agonism. GIPR agonists, once chemically integrated with GLP1R agonism, have demonstrated metabolic benefits and reduced body weight in mice when compared with pharmacokinetically matched GLP1R agonists^122,189^. There are multiple reasons why GIP agonism might provide supplemental metabolic benefits to GLP1 therapy, apart from lowering body weight and food intake via GLP1R-independent mechanisms^184,185^. GIP blocks the emetic effects of GLP1R agonism in musk shrews^190^ and near-normalization of blood glucose has been reported to restore the insulinotropic effect of GIP in patients with T2D^191^. Furthermore, GIP agonism enhances adipocyte storage capacity to protect from adipocyte lipid spill over and ectopic lipid deposition^192^. Nonetheless, as discussed in the preceding subsection, the use of GIPR agonists for the treatment of obesity and T2D is controversial.

Importantly, phase II results for two unimolecular, long-acting GIPR/GLP1R co-agonists have been reported. The first, NN9709 (formerly MAR709 and RG7697) (Table 2), is suited for once-daily subcutaneous injection and demonstrates balanced high potency at human GLP1R and GIPR^193^. NN9709 reduced blood glucose, body weight and total cholesterol in a 12-week phase II study of T2D as compared with placebo^193^. However, the improvement in body weight was not statistically different relative to dose-titrated liraglutide. Development of this specific co-agonist was discontinued in 2020 given the efficacy of semaglutide 2.4 mg in phase III clinical trials (see Related links). More recently, in mice with CNS deletion of GIPR, MAR709 was shown to lose its superior ability to lower body weight and food intake relative to a pharmacokinetically matched GLP1 (ref.^185^). This observation underscores the contribution of central GIPR agonism to the body weight-lowering mechanism of this AOM.

Tirzepatide (formerly LY3298176) possesses fivefold increased relative potency at human GIPR as compared with GLP1R and is designed for once-weekly subcutaneous injection^122^. In a phase II trial in patients with T2D, 26 weeks of treatment with tirzepatide demonstrated dramatically superior results relative to a specific once-weekly GLP1R agonist in both HbA_1c_ and body weight lowering^194^. At the highest doses employed, glucose control was unusually strong with nearly one third of patients achieving HbA_1c_ <5.7%, and weight loss in these patients with diabetes exceeded 10%. Collectively, these results have generated great interest in GIPR/GLP1R co-agonists, while deepening the debate as to the relative direct and indirect contribution of GIPR agonism^192,195,196^. In a recent phase III trial in patients with T2D of excess weight, tirzepatide showed superior ability to decrease HbA_1c_ and body weight at all doses tested, relative to treatment with semaglutide 1 mg (ref.^125^). Forty weeks of treatment with tirzepatide decreased HbA_1c_ <5.7% in 29–51% of patients relative to 20% treated with semaglutide, and decreased body weight ≥15% in 15–40% of patients relative to 9% with semaglutide^125^. A subsequent phase III trial in patients with obesity or overweight with diabetes confirmed that treatment with tirzepatide for 40 weeks similarly decreased HbA_1c_ <5.7% in 34–52% of patients and lowered body weight ≥15% in 13–27% of patients^127^. Consistent with this, in patients with T2D who are insulin-dependent, 1 year of treatment with tirzepatide improved glycaemic control with much greater efficacy relative to comparative treatment with insulin (degludec)^126^. How the more recently approved semaglutide 2.4 mg compares with tirzepatide remains to be determined^35–38^.

Co-therapy of GLP1R agonism with glucagon (GcgR) agonists is designed to employ more than a single mechanism in body weight reduction (appetite suppression, thermogenesis and lipolysis, respectively), while minimizing the risk of hyperglycaemia^186,197^. Clinical results have been reported for two GLP1R/GcgR co-agonists (cotadutide, formerly MEDI0382 and SAR425899). Each of them is palmitoylated, with once-daily time action notably more potent at GLP1R relative to GcgR. In a 54-week phase IIb study in patients with overweight and obesity with T2D, cotadutide reduced body weight and hepatic fat content and improved glucose tolerance relative to placebo^198^. Mean weight loss was ~5%, with 15.5% of patients achieving weight loss greater than 10% relative to 5.8% receiving liraglutide 1.8 mg. SAR425899 has completed phase I trials in healthy volunteers and patients with T2D^199–201^. Body weight loss of ~7% was reported after 4 weeks of treatment, with improvements in glucose tolerance. Whether additional unimolecular GLP1R/GcgR co-agonists with greater relative glucagon activity or more extended duration of action prove more effective, and sufficiently safe for chronic use, remains to be determined^202^.

Given the power of the approach, multi-agonism therapy has been repeatedly employed in preclinical treatment of obesity, typically but not exclusively in combination with some form of GLP1 agonism. Representative co-therapies include leptin with pramlintide^180–182,203,204^, leptin with exendin 4 or FGF21 (ref.^205^), salmon calcitonin with exendin 4 (ref.^206^), GLP1 with PYY^207^, exenatide with CCK^208^ and liraglutide with setmelanotide^209^.

Further development specific to glucagon-like peptides has been anchored by the enhanced performance demonstrated for GLP1 co-agonists with GIP or glucagon agonism. These results have promoted integration of the three activities into a single-molecule tri-agonist that includes balanced and full agonism at GLP1R, GIPR and GcgR. Such a tri-agonist has shown great promise in animal testing and advanced to clinical studies^210,211^. The presence of both GLP1 and GIP components within the same molecule is reported to more effectively minimize the risk of glucagon-mediated hyperglycaemia, and thereby permit more aggressive dosing to achieve additional weight reduction.

In 2015, the first report of superior reductions in body weight and plasma cholesterol in DIO mice as compared with placebo, a GLP1R mono-agonist and a matched GLP1R/GIPR co-agonist, were disclosed for a specific tri-agonist^210^. The contribution of each individual receptor activity within the tri-agonist was further identified through testing in receptor knockout mice, and with selective chemical antagonism at each receptor. NN9423, a peptide tri-agonist, has advanced to clinical study, but outcomes have yet to be reported. Additional drug candidates include a series of fatty acylated unimolecular GLP1R/GIPR/GcgR tri-agonists (see Related links) and an Fc fusion^183^. The latter of these candidates (HM15211) is currently in early clinical trials for treatment of nonalcoholic steatohepatitis (Table 2). LY3437943 (GGG) is a GIP/GcG/GLP1 tri-agonist suitable for once-weekly dosing. In phase I clinical trials, 12 weeks of treatment in patients with T2D revealed substantially greater weight loss relative to treatment with tirzepatide but, importantly, equal glycaemic efficacy^212^.

### Leptin, leptin sensitizers and MC4 agonists

The discovery of leptin in 1994 (ref.^47^) forged our understanding of how peripheral hormones signal to the brain to regulate energy balance (Box 1; Fig. 2). The loss of leptin leads to severe metabolic disturbances, which include extreme hyperphagia, lipodystrophy and hypothalamic amenorrhoea^136,213^. Several clinical studies confirmed the effectiveness of rDNA-derived human leptin for the treatment of hypothalamic amenorrhoea^214,215^ and leptin supplementation in *ob/ob* mice is sufficient to restore fertility^216^. Metreleptin (Amylin Pharmaceuticals, now AstraZeneca) was approved by the FDA in 2014, and by the European Medicines Agency (EMA) in 2018, for the treatment of lipodystrophy, and leptin supplementation largely normalizes metabolic and neuroendocrine alterations in humans with congenital leptin deficiency^95,118,136,217^ and in patients with anorexia nervosa^218^. However, although leptin supplementation is effective in individuals with congenital leptin deficiency, the hormone shows little ability to lower body weight under conditions of common, polygenetic, obesity^115,116,137,138^. Also, despite not being correlative to lower efficacy or safety, the development of antibodies against metreleptin constitutes an obstacle for its clinical use^219^. Whereas leptin appears not to hold promise as a stand-alone therapy for the treatment of common obesity, its combination with pramlintide (Amylin Pharmaceuticals) induces greater body weight loss in individuals of excess weight relative to treatment with either drug alone^181,220^. Improvement of leptin responsiveness has also been confirmed preclinically following co-therapy with either exendin 4 (ref.^205^), FGF21 (ref.^205^) or GLP1/glucagon^221^. Also, plant-derived small molecules such as celastrol^222^ and withaferin A^223^ have been shown to decrease body weight through improvement in leptin sensitivity (Table 2).

Leptin regulates energy metabolism via activation of POMC neurons in the ARC while, at the same time, inhibiting AgRP neurons in the same area (Box 1; Fig. 2). POMC neurons project to the paraventricular nucleus (PVN), where they induce satiety through activation of the brain MC4R. Although the brain MC4R is an acknowledged target for the treatment of obesity, the development of selective and safe MC4R agonists imposes notable challenges. Over the last 30 years, a series of MC4R agonists have been developed and shown to decrease body weight and food intake in experimental DIO animals^224^. However, MC4R agonists are prone to cross-stimulate the structurally related MC1, MC3 and MC5 receptors, which serve important roles in various neuroendocrine processes including hair and skin pigmentation, energy homeostasis and erythrocyte differentiation. Furthermore, activation of MC4R can elevate blood pressure and heart rate through activation of the sympathetic nervous system and induce sexual arousal in males^224,225^. MC4R agonists that were clinically tested but stopped for insufficient weight loss or the aforementioned adverse effects include LY2112688 (Eli Lilly), MC4-NN-0453 (Novo Nordisk), MK-0493 (Merck) and AZD2820 (AstraZeneca)^224^. By contrast, setmelanotide is a structurally related MC4R agonist developed by Rhythm Pharmaceuticals. Unlike previous MC4R agonists, setmelanotide did not affect heart rate and blood pressure in monkeys and humans^224,226,227^. This peptide exhibited profound weight loss in humans with congenital deficiency of either POMC^228^ or LEPR^229^ and was well tolerated without any major adverse effects in phase III clinical trials^230^. The FDA approved setmelanotide in November 2020 for the treatment of obesity in patients with POMC, PCSK1 or LEPR deficiency. Future studies are warranted to investigate whether setmelanotide can decrease body weight in more common, polygenetic forms of obesity. Studies in patients with Prader–Willi syndrome have demonstrated that setmelanotide can decrease body weight in individuals where the primary source of obesity is not directly attributable to the melanocortin system.

### Amylin

Amylin (also known as IAPP) is a peptide that is co-secreted with insulin and reduces food intake through central control of satiety pathways^231,232^ (Box 1; Fig. 2). Amylin activates specific receptors including those of the calcitonin gene-related peptide (CGRP). Although the major effect of amylin on energy metabolism is mediated through increasing satiety, amylin has also been shown to affect hedonic control of eating, including a reduction in feeding reward neurocircuits^233^. However, the clinical application of native amylin in treating obesity has been shadowed by physical aggregates related to pancreatic islet death in humans^234^, a finding not observed with rat amylin^235^. The anorexigenic potential of amylin promoted the development of pramlintide, a rat-based synthetic analogue of amylin^236^.

Pramlintide is approved by the FDA for use in patients with T1D and T2D who are using mealtime insulin alone, or in combination with an oral agent such as metformin or a sulfonylurea^165,237^. Importantly, effects of pramlintide on reducing food intake and body weight are not limited to patients with impaired glucose metabolism^233^. Therefore, other amylin analogues with improved pharmacokinetics are being considered as AOMs. Amylin agonists seem to be particularly useful for weight loss in combination with other agents, such as leptin^181,220^ or calcitonin receptor agonists^238^.

The human amylin receptor subtypes are complexes of the calcitonin receptor with receptor activity-modifying proteins^239^. Recently, dual-acting amylin and calcitonin receptor agonists (DACRAs) have been developed as potential AOMs (Table 2). Several DACRAs (for example, davalintide (AC2307), KBP-088, KBP-089, KBP-042) have been shown to induce weight loss in animal models of obesity^165,240–242^. In addition, a long-acting amylin analogue, cagrilintide, suitable for once-weekly treatment has successfully completed a phase Ib trial (Table 2) and is favourably progressing in subsequent studies in combination with semaglutide to what might constitute enhanced chronic efficacy^243^.

### Ghrelin

As a peptide hormone secreted from x/a-like cells (P/D1 cells in humans) of the gastric fundus, ghrelin acts on hypothalamic feeding centres to stimulate food intake^244^ (Fig. 2). Independent of its orexigenic effect, ghrelin promotes adiposity and elevates blood glucose through inhibition of insulin secretion^245^. Envisioned strategies to harness ghrelin biology for potential treatment of obesity include suppression of active circulating hormone and antagonism of signalling at its receptor, the growth hormone secretagogue receptor (GHSR). The latter can be achieved through GHSR antagonists and inverse agonists, such as the liver-enriched antimicrobial peptide 2 (LEAP2), or the des-acyl form of ghrelin (DAG). Therapeutic interest has been spurred by observations in rodents, where neutralization of acyl-ghrelin^246^, inhibition of ghrelin *O*-acyltransferase (GOAT) as the activating fatty acylation enzyme^247^ or direct antagonism of GHSR^248^ have demonstrated decreases in body weight and food intake.

In patients with Prader–Willi syndrome, circulating levels of acyl-ghrelin are increased^249^ and 14-day treatment with a UAG analogue (AZP-531) (Table 2) improved food-related behaviour, body fat mass and postprandial levels of blood glucose, without any major sign of intolerability^250^. Nonetheless, ghrelin is a disputed target for treatment of obesity^251,252^, where the circulating concentrations of acyl-ghrelin are reported to be elevated in individuals who are lean and those with anorexia, and low in certain conditions of obesity. Furthermore, excess body fat is associated with ghrelin unresponsiveness. This is potentially mediated by a LEAP2-associated increase in obesity that serves to competitively bind GHSR and inhibit biological signalling^253^.

Approaches to decrease acyl-ghrelin include a therapeutic peptide vaccine that ameliorated body weight gain in rodents, interestingly without affecting food intake. The efficacy was reported to be specific to the plasma binding of the acyl form of ghrelin^254^. A similar acting vaccine, CYT009-GhrQb (Table 2), was developed by Cyto Biotechnology. The vaccine advanced to early clinical trials (phase I/II) in which it showed no effect on body weight or food intake^255^. Separately, no long-term beneficial effects on body weight or food intake were reported when a specific anti-ghrelin monoclonal antibody was tested in DIO mice at Amgen^256^. A comparable outcome resulted in the use of anti-ghrelin Spiegelmers developed at NOXXON Pharma that only moderately improved metabolism in preclinical studies, with no effect on food intake after 8 days of treatment^246^.

In summary, pharmacotherapies targeting the ghrelin pathway so far have yet to reveal a clinically validated AOM candidate. Targeting the ghrelin pathway, however, warrants further investigation as ghrelin remains the only known circulating signal to increase hunger and potently activate hypothalamic AGRP neurons that drive appetite^244^.

### Targeted mitochondrial uncouplers

The tissues most involved in thermogenesis are skeletal muscle and adipose tissue, most notably brown adipose tissue. Energy derived from dietary substrates is captured by TCA-mediated catabolism in the mitochondria in association with an electron transport chain leading to ATP synthesis^257^. UCP1, localized in the inner mitochondrial membrane of brown and beige adipocytes, catalyses the transport of protons across the mitochondrial membrane and, thereby, induces mitochondrial uncoupling of oxygen consumption from ATP synthesis^258,259^. Pharmacologically, UCP1 activity can be induced by catecholamines with subsequent activation of β_3_-adrenergic receptors of brown adipose tissue^257^. Thyroid hormone (T3) is an endogenous entity with uncoupling capability mediated by several different mechanisms^260^.

Enhancement in mitochondrial uncoupling can have beneficial health effects. Mitochondrial uncouplers, such as 2,4-dinitrophenol (DNP), increase mitochondrial inefficiency, rendering metabolism and production of ATP less efficient^261^. Although DNP was welcomed for obesity treatment in 1934 (ref.^55^), it was later banned from therapeutic use due to multiple adverse effects and numerous reports of DNP-associated deaths^261^. Nonetheless, the substance has continued to be used by bodybuilders and others. The UK Food Standard Agency issued a warning in 2003, given increasing concern for toxicity associated with unregulated use that DNP was ‘not fit for human consumption’ (see Related links).

Mitochondrial uncouplers are cytotoxic at high concentrations, an effect resulting from a drop in ATP concentration and on plasma and lysosomal membrane depolarization and permeabilization. However, the effect is concentration-dependent, and at doses that are not toxic, mitochondrial uncoupling can protect cells against death^262^. Consequently, the development of mitochondria-specific and safer uncoupling agents suitable for human use might yet result in a powerful and differentiated approach to treating these diseases^263^. Recent studies using a controlled-release oral formulation of DNP, called CRMP (controlled-release mitochondrial protonophore), is one prominent attempt to achieve an enhanced therapeutic index. In rats, CRMP was employed to achieve low-level hepatic mitochondrial uncoupling that reversed hypertriglyceridemia, insulin resistance, hepatic steatosis and diabetes^264^.

BAM15 ((2-fluorophenyl){6-[(2-fluorophenyl)amino](1,2,5-oxadiazolo [3,4e] pyrazin-5-yl)} amine) (Table 2) is a novel mitochondria-specific protonophore uncoupler that demonstrates similar potency to DNP to increase energy expenditure^265^. BAM15 is an orally administered drug that can increase nutrient oxidation, and decreases body fat mass without altering food intake, lean body mass, body temperature or haematological markers of toxicity. Mice treated with BAM15 were reported to be resistant to weight gain^265,266^. BAM15 improves insulin sensitivity in multiple tissues and in vitro enhanced mitochondrial respiration, improved insulin action and stimulated nutrient uptake by sustained activation of AMPK. These results collectively illustrate that mitochondrial uncoupling with BAM15 has robust anti-obesity and insulin-sensitizing effects, without compromising lean mass or affecting food intake^265,266^. However, it remains too early to say with confidence whether BAM15 or another related approach will provide much-enhanced therapeutic safety for treatment of obesity-related comorbidities or excess body weight itself.

### GDF15

Macrophage inhibitory cytokine 1 (MIC1; also known as GDF15) has gained attention as a target for obesity treatment^267^. GDF15 is a divergent member of the transforming growth factor-β (TGFβ) superfamily^267^. Physiologically, GDF15 is expressed in multiple tissues at a low concentration, but increases in response to or association with tissue injury, cancer, metabolic disease, CVD and inflammation^267,268^. GDF15 has also been proposed to act as an anti-inflammatory cytokine in the infarcted heart^269^.

Exogenous administration of rDNA-derived GDF15 and analogues decreases body weight in diet-induced obese mice and non-human primates, suggesting a homeostatic role in energy homeostasis^267,270^. Recently, GDF15 was shown to physiologically regulate energy homeostasis and body weight — primarily via appetite suppression — through activation of the receptor, GDNF family receptor α-like (GFRAL)^270^. Some studies suggested that the anorectic effect of GDF15 is mediated through induction of nausea and engagement of emetic neurocircuitries^271,272^, but this has not been confirmed by all studies^270^. Nonetheless, its depletion results in increased body weight^273,274^, whereas GDF15 overexpression has the opposite effect^274–276^. Chronic study demonstrating sustained efficacy, sufficiently devoid of safety risks such as nausea/vomiting, tumorigenicity and cachectic lean body mass reduction, needs to be thoughtfully considered. Ultimately, only in human study can the assessment of whether GDF15 analogues will prove efficacious and safe for weight loss management be determined^267^.

### Peptide tyrosine tyrosine

Peptide tyrosine tyrosine (PYY) is a member of the NPY family that is co-secreted from the intestinal L cells as PYY_1–36_, along with GLP1. After being released, PYY_1–36_ is rapidly cleaved by DPP-IV to its major active form, PYY_3–36_, a high-affinity agonist at the NPY receptor type 2 (Y2R). This receptor is highly expressed in parasympathetic and sympathetic neurons of the periphery as well as in several regions of the CNS, including the limbic and cortical areas and the brainstem^277^. In the hypothalamus, Y2R is highly expressed on NPY neurons of the ARC^278^ and PYY_3–36_ decreases food intake and body weight in rodents^278–280^ and humans^207,281,282^, at least in part through its ability to silence Npy neurons and, hence, to indirectly activate Pomc neurons^278^. Additional mechanisms that may be implicated in the regulation of food intake by PYY_3–36_ include Y2R-mediated activation of the mesolimbic dopaminergic system as well as of GABAergic and glutamatergic neurons in cortical and subcortical regions and the brainstem^277^. Consistent with the relevance of dopaminergic signalling in mesolimbic brain regions, PYY_3–36_ has effects that go well beyond the regulation of food intake, and include memory and learning, central information processing and behavioural response to dopamine-stimulating drugs^277^. The ability of PYY_3–36_ to decrease food intake in rodents and humans has stimulated the development of PYY_3–36_ analogues for the treatment of obesity^283^. In line with this notion, several long-acting PYY_3–36_ analogues (NN9748 and NNC0165-1875) have completed phase I trials for the treatment of obesity (see Related links) and NNC0165-1875 is now being assessed in a phase II combination study with semaglutide (see Related links). Additionally, Lilly Research Laboratories announced a phase I trial with a PYY analogue for the treatment of T2D (see Related links).

## Outlook and future directions

The pursuit of AOMs has been a long-standing endeavour propelled in recent years by several concurrent developments. These include the dramatic increase in the global prevalence of obesity, the significant advances in molecular understanding of appetite homeostasis along with the identification of several novel drug targets, as well as the success in developing incretins as drugs for T2D that has provided unprecedented efficacy in body weight management. It seems plausible that a 20% or greater reduction in body weight may yet be possible based on late-phase clinical reports. If so, it is interesting to ponder whether patients of far higher initial body weight might find the next 20% reduction to be easier or harder to achieve in a relative sense, as these are the individual subjects of greatest need.

GLP1R agonism is establishing a heightened foundation for measuring performance with other entities, and the full depth of its efficacy and the ability to chronically sustain weight loss in multiple populations, many distinct from those in which initial drug registration has occurred, remains to be determined. As with any rapidly advancing field, there are more questions than answers. Of primary interest is why GLP1R agonism works so well and how GIP might synergize with GLP1 to enhance weight loss. Short of the results that have been achieved in vivo, most notably the 6-month and 1-year clinical studies that appear to indicate significant additional benefits of semaglutide when compared with liraglutide, it is difficult to ascribe a molecular basis for that difference. These two agents are both highly potent and selective GLP1R agonists, similarly fatty acylated, that provide sustained drug plasma concentrations when used as prescribed. The difference is not simply a matter of extended time action as even a long-action Fc agonist such as dulaglutide does not match the body weight lowering of semaglutide^284^. Initial study suggests increased activity in central locations of importance to weight control^123^. However, this is just a beginning and a deeper molecular understanding might lead to even further improvements in GLP1R agonists, or other agents that might act by an independent mechanism at similar anatomical sites.

Unquestionably, the clinical results with tirzepatide have captured great attention and fuelled interest in GIP-based dual agonists and other combinatorial approaches. However, is this interest justified by these clinical results? The situation appears to exemplify that despite the enormous advance in our molecular understanding of obesity, we remain relatively primitive in ascribing in vivo efficacy to mechanism. It remains to be demonstrated in mechanistic detail how GIPR agonism serves as the basis for the heightened efficacy of tirzepatide relative to dulaglutide. Very recently, it was shown that CNS loss of GIPR renders mice resistant to GIP-induced body weight loss, indicating that GIP regulates energy metabolism via CNS GIPR signalling^185^. Substantiating the relevance of this finding, it is noteworthy that the superior weight-lowering effect of MAR709 relative to a GLP1 monotherapy of matched structure and pharmacokinetics vanished in CNS *Gipr* knockout mice^185^. The central mechanisms and target regions for GIP synergy with GLP1 remain to be determined, and notably there are conflicting preclinical results that promote GIPR antagonism as a therapeutic option for treating obesity^184^. In time, these questions and uncertainties will eventually be answered.

Next-generation discoveries are heavily influenced by current clinical performance and limitations in our ability to successfully translate in vitro and animal pharmacology to human experiments. High-dose semaglutide and tirzepatide are reporting sustained reduction in body weight of approximately 0.5 kg per week. This is a breakthrough performance relative to registered AOMs that begs the question of what the highest next priority is, and whether we have the skills necessary to properly achieve it. Clearly, additional mechanisms of action that can match the performance of these two drugs would be welcomed, but to document this requires appreciably long studies. Underpowered 4-week, 6-week, 8-week and, even, 12-week studies without suitable registered drugs as controls have largely failed to document relative efficacy.

Efficacy studies struggle with the question of how much additional weight reduction is advisable in a finite period, and the duration necessary for documenting it with confidence. Given the efficacy that is being achieved and the chronic nature of obesity, it is arguable that maintaining the rate in weight loss for subjects of continued excess weight is the primary objective. These studies are lengthy and rarely undertaken until there is great confidence for success. Shortening the studies with the objective of accelerating the relative rate of weight reduction may not prove advisable for the patient and could lead to adverse effects that eliminate approaches that otherwise would prove viable, if applied less aggressively. This is a point of particular importance in the assessment of glucagon-based tri-agonists that aim to outperform GLP1–GIPR co-agonists, as glucagon is likely an agonist of reduced therapeutic index relative to the two incretins.

In a related manner, might drug candidates that fail in monotherapy prove successful when added to the best-in-class incretins either at initiation of therapy or after sizeable weight loss? The clinical success of GLP1 with GIP raises the question of whether adjunctive therapy of semaglutide with another weight-lowering agent such as amylin, PYY or FGF21 can safely lower body weight beyond what is possible with either drug alone. In this regard, it should be noted that leptin therapy proved successful in reducing body weight when used following sizeable weight loss in obese mice^181,205,221^. Might the same prove true in selected patients with obesity now that comparable percent reductions in body weight with what has proven successful preclinically are being achieved with semaglutide and tirzepatide?

Finally, there is the question of what is most needed to accelerate the realization of the next leap forward in safely normalizing body weight. Next-generation multi-omics have provided some novel targets, but, overall, rapidly evolving enabling technologies have been more useful in characterizing preclinical mechanism of action than in discovery of clinically successful drug candidates. Iterative rodent testing largely using diet-induced obese mice and rats has been the primary screen to assess body weight lowering. Genetic models and, even more so, engineered mice where specific receptors have been deleted, and increasingly so in a target-specific manner, have proven of indispensable value to investigation of mechanism of action.

The clinical situation is more challenging, where there is infrequent access to individuals homozygous-deficient in a specific biological mechanism. In those rare instances, the nature of the obesity and the response to therapy differ from the general population. Additionally, selective antagonists suitable for pharmacological use are seldom available to selectively silence a single mechanism of action to explore its relation to endogenous control of body weight, or to block the action of a specific drug or a single element in a multi-action peptide, such as the incretin co-agonists. Lastly, the simultaneous comparison of peptides matched in structure and pharmacokinetics, but otherwise devoid of a single biological activity, constitutes a prohibitive investment when the length of study is measured in months. Consequently, what we most need to speed drug discovery and optimization is correlative diagnostic means to complement a body weight scale. If we could serologically or non-invasively predict with increased confidence those patients and mechanisms that are likely to succeed long term, this would promote better outcomes and increase exploratory clinical research to identify molecular entities and combinations that most justify assessment in long-term studies. In analogy, it is readily recognized what plasma glucose monitoring and HbA_1c_ have meant to diabetes care and drug discovery relative to urine testing or monitoring of longer-term microvascular outcomes. If a predictive correlate between metabolic profiling and propensity to weight loss can be established, this could have a profound influence on the future of healthcare in obesity.

## Summary

Pharmacological management of obesity has a lengthy history populated with multiple prominent disappointments. The basis of failure has been multifactorial and pertains to the limited translational value of animal models to predict cardiovascular safety coupled with considerable patient heterogeneity. Patients with obesity are often at high risk for vascular diseases and afflicted with comorbidities that complicate assessment of drug safety. Long-term, large-scale clinical trials in heterogeneous patients with obesity are expensive to conduct and difficult to justify when success has been so elusive and failures so prominent.

The recent precedent-setting results with semaglutide and tirzepatide, in which each reported mean weight loss well in excess of 10%, employing a GLP1 mechanism that has separately proven to improve cardiovascular outcomes in T2D studies, inspires confidence for the future. Clinical application will continue and focus on relative efficacy and safety, which is difficult to ascribe when best-in-class candidates are simultaneously rapidly advancing and not immediately accessible for direct comparative clinical study^125^. Independently, setmelanotide and leptin have proven successful in obesity management of individuals with congenital deficiency in genes of the leptinergic–melanocortinergic pathway. These successes illuminate the paths for future research targeting other monogenetic forms of the disease and the possibility for additive pharmacology in broader populations of patients with obesity. A more thorough characterization of patients should serve to increase the near-term likelihood for success and provide informed direction for advancing the next generation of AOMs. Ongoing clinical studies will determine whether more efficacious drugs than semaglutide and tirzepatide might achieve efficacy comparable with bariatric surgery. The many prospects currently being considered suggest that one or more might achieve this lofty objective.

## Glossary

Obesity: Excess body fat mass as a result of a chronic surplus of energy intake over energy expenditure.
Diabetes: Chronically elevated blood glucose as a result of insufficient action or production of insulin.
Anti-obesity medications: (AOMs). Pharmacotherapy aiming to decrease excess body fat.
Incretin: An intestinal peptide that stimulates the release of insulin.
Poly-agonists: Unimolecular peptides capable of acting on multiple receptors.
